# Triple antibody-associated autoimmune encephalitis overlap: a case report of co-existing MOG-IgG, anti-NMDAR, and anti-mGluR5 positivity

**DOI:** 10.3389/fimmu.2026.1848797

**Published:** 2026-05-22

**Authors:** Chao Zhao, Jing Liu, Laiyun Zhu, Xueqin Zhang, Nuan Wang

**Affiliations:** 1 Department of Neurology, The Affiliated Xuzhou Municipal Hospital of Xuzhou Medical University, Xuzhou, China; 2 Suzhou Medical College of Soochow University, Suzhou, China; 3 Xuzhou First People’s Hospital, Xuzhou, China

**Keywords:** autoimmune encephalitis, metabotropic glutamate receptor type 5, myelin oligodendrocyte glycoprotein, N-methyl-D-aspartate receptor, overlap syndrome

## Abstract

**Background:**

Autoimmune encephalitis (AE) refers to a group of inflammatory brain disorders mediated by autoantibodies against neuronal surface antigens, synaptic receptors, or glial proteins. The co-occurrence of anti-N-methyl-D-aspartate receptor (NMDAR) encephalitis and myelin oligodendrocyte glycoprotein (MOG) antibody-associated disease in a single patient is rare. Cases with concurrent anti-mGluR5 antibodies are even rarer, with only one previously reported globally in 2022.

**Case presentation:**

A 20-year-old Chinese man presented with fever, headache, progressive cognitive impairment, and psychiatric symptoms, but no seizures. Cell-based assays (CBA) showed positive anti-NMDAR IgG in both serum (1:30) and cerebrospinal fluid (CSF, 1:100), positive MOG antibodies (serum 1:100, CSF 1:100), and low-titer anti-mGluR5 IgG positivity (serum and CSF both 1:10). He received intensive multimodal immunotherapy, including high-dose methylprednisolone, intravenous immunoglobulin, plasma exchange, and rituximab. The patient achieved complete clinical recovery within three months. At the four-month follow-up, mGluR5 antibodies had become negative, while MOG and NMDAR antibody titers were markedly reduced (both from 1:100 to 1:10). Tumor screening was negative.

**Conclusions:**

This is the first report of a seizure-free, triple-positive autoimmune encephalitis subtype presenting with prominent cognitive impairment, thereby expanding the clinical spectrum of recently described overlapping autoimmune encephalitis syndromes. The low titer and rapid seroreversion of mGluR5 antibodies suggest a possible bystander phenomenon secondary to blood-brain barrier disruption.

## Introduction

Anti-N-methyl-D-aspartate receptor (NMDAR) encephalitis is the most common form of autoimmune encephalitis (AE), accounting for 54-80% of cases (1). Myelin oligodendrocyte glycoprotein antibody-associated disease (MOGAD) is an inflammatory demyelinating disorder of the central nervous system (CNS). It can overlap with AE, forming a distinct overlap syndrome known as MNOS (2), which is characterized by seizures, psychiatric symptoms, and demyelinating events. The underlying mechanism may be related to the co-expression of NMDAR and MOG on oligodendrocyte processes (3). In contrast, metabotropic glutamate receptor 5 (mGluR5) antibody-associated encephalitis is extremely rare. mGluR5 belongs to the G protein-coupled receptor family (4) and is primarily expressed at postsynaptic terminals of neurons and microglia. This disease entity was first reported in 2011 in two patients with limbic encephalitis associated with Hodgkin lymphoma (5). Clinically, the coexistence of mGluR5 antibodies with other autoantibodies is very uncommon. It was not until 2022 that Fu et al. reported the first case of mGluR5 encephalitis presenting as meningoencephalitis with additional antibody positivity (6). To date, no cases have been reported with the coexistence of NMDAR, MOG, and mGluR5 antibodies presenting predominantly with cognitive impairment. Here, we report a rare case of triple-antibody overlap syndrome. Unlike typical MNOS, this patient experienced no seizures throughout the disease course and instead exhibited a distinctive clinical phenotype dominated by cognitive decline. This finding significantly expands the recognized clinical spectrum of such rare overlap syndromes.

## Case presentation

A 20-year-old Chinese man presented with headache, fever, and memory decline for one week. He had no prior history of neuropsychiatric disorders, and his family history was unremarkable. The patient developed intermittent fever and headache following a common cold on November 7, 2025, with a maximum temperature of 39 °C. A cranial CT scan performed on November 18, 2025, showed no significant abnormalities. Treatment with ceftazidime and dexamethasone relieved the headache, but the intermittent fever persisted.Although the headache improved with this regimen, intermittent fever persisted.Two days later, on November 20, 2025, the patient’s condition took a notable turn: he began exhibiting memory impairment, behavioral abnormalities, personality changes and his Mini-Mental State Examination(MMSE) was 13/30— a clinical evolution that raised strong suspicion for autoimmune encephalitis and prompted transfer to our department.

### Laboratory investigations upon admission

were notable for peripheral leukocytosis (18.27 × 10^9^/L). Routine biochemical tests, antinuclear antibody panel, and thyroid function tests were all within normal limits. Screening for HIV and syphilis (rapid plasma reagin)was non-reactive. To exclude paraneoplastic etiology, chest CT and abdominal ultrasound were performed, neither of which revealed any definite masses or suspicious lesions.

### Neuroimaging and neurophysiology

**Figure 1:**
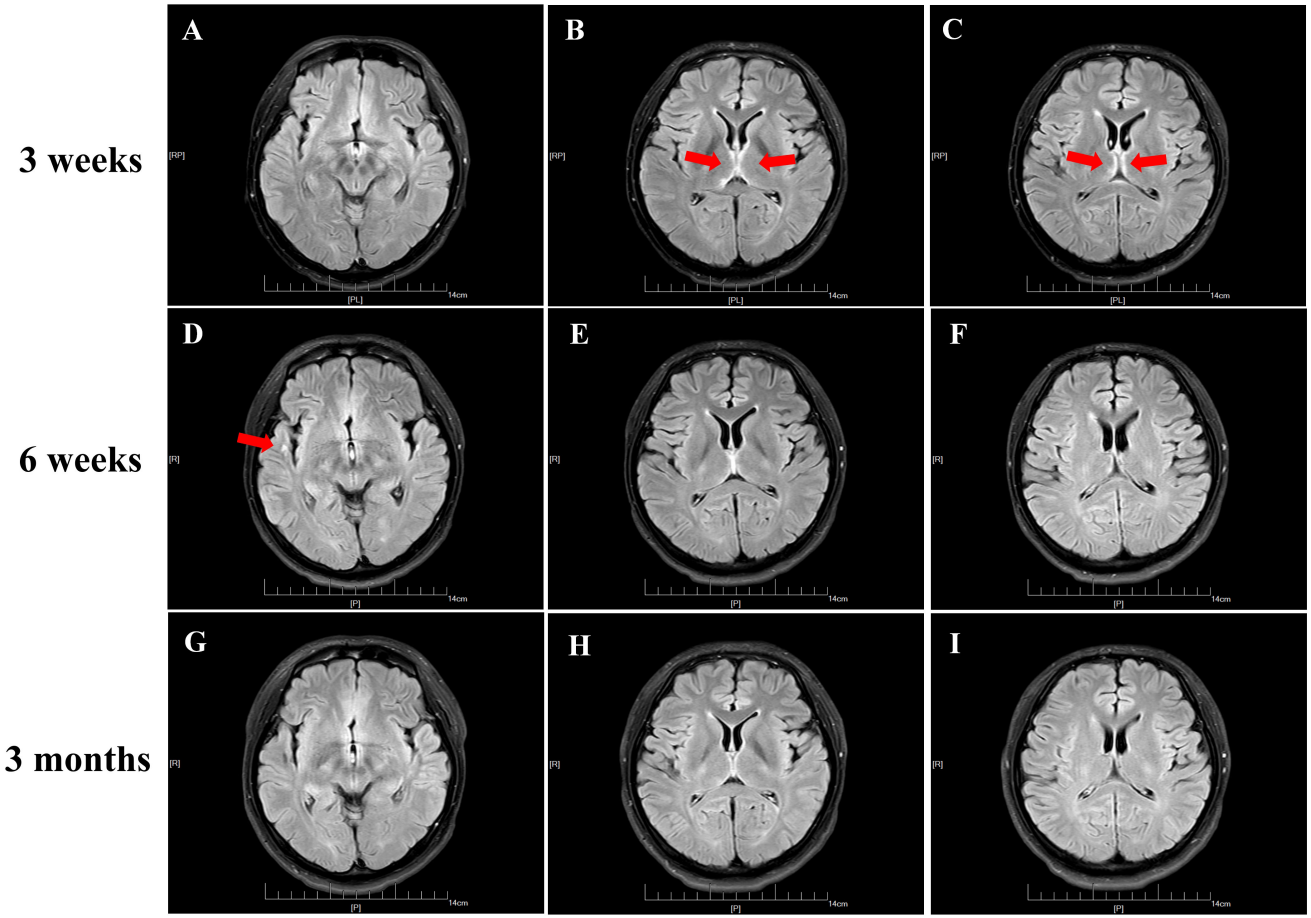
**(A–C)** Brain MRI of the patient at three weeks after the symptom onset showed hyperintensity of the extensive cortex in bilateral thalamus on fluid attenuated inversion recovery (FLAIR) imaging. **(D–F)** MRI was repeated at six weeks and showed a significant improvement of the imaging abnormality hyperintensity in bilateral thalamus, but hyperintensity in Right temporal lobe. **(G–I)** MRI performed at almost three months after initial symptom onset depicted no signal abnormalities on FLAIR sequences.

Brain MRI showed subtle FLAIR hyperintensities in the bilateral thalamus and the right hippocampus (**Figure 1**). Continuous EEG monitoring revealed diffuse low-amplitude theta activity (4–7 Hz), a pattern consistent with non-specific encephalopathy rather than epileptiform discharge.

### Cerebrospinal fluid analysis

**Figure 2:**
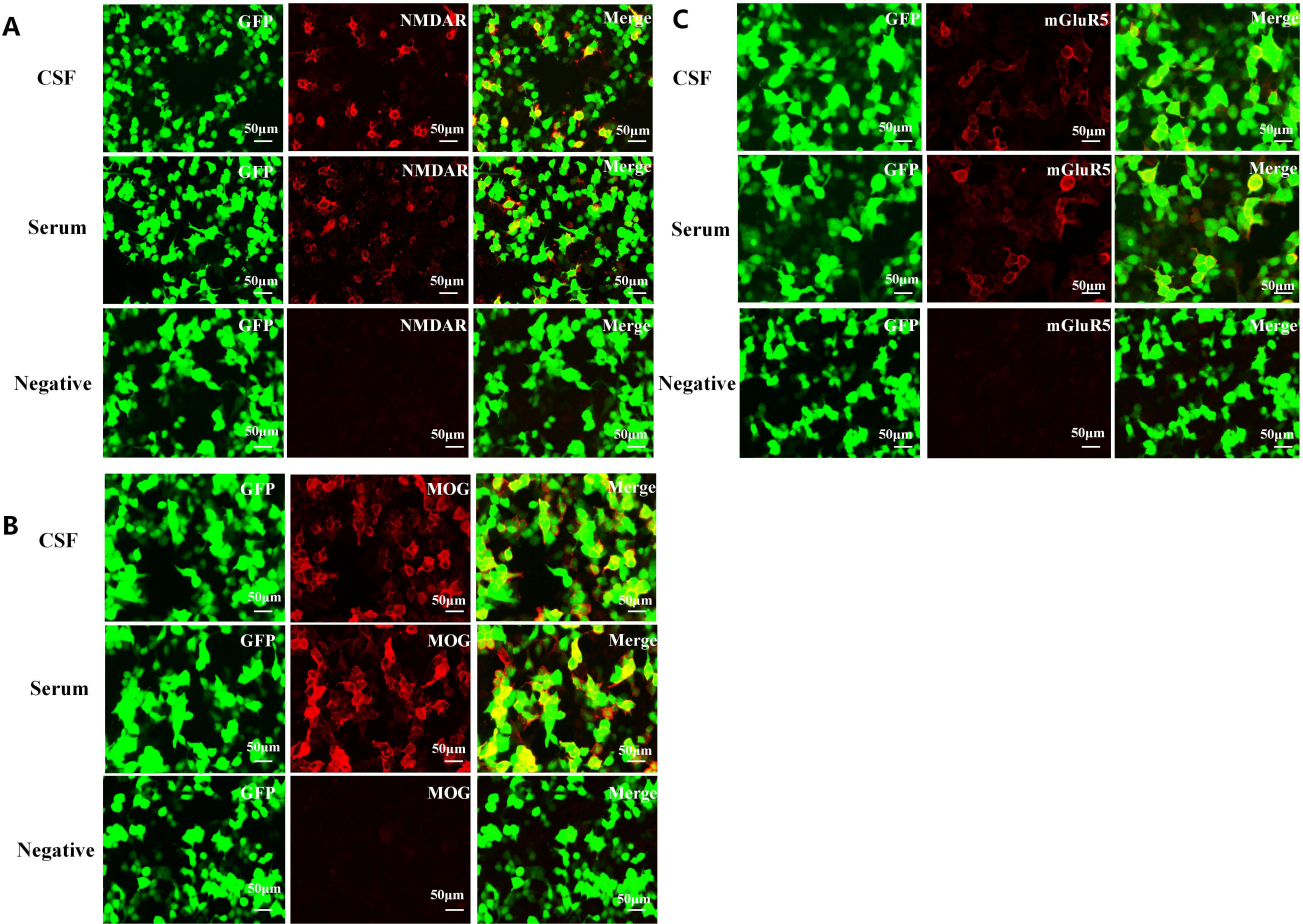
Immunofluorescence of anti-NMDAR, anti-mGluR5 and MOG antibodies in the patient's cerebrospinal fluid and serum. These antibodies bound on the antigens expressed by the HEK293 cells and visualized by the immunofluorescence antibody. **(A)** A cell-based assay (CBA) demonstrates positive NMDAR (1:100, 1:30), MOG (1:100, 1:100) and mGluR5 (1:10, 1:10) expression in both CSF and serum. **(A)** NMDAR antibody; **(B)** MOG antibody; **(C)** mGlur5 antibody.

revealed an elevated opening pressure (180 mmH_2_O) and marked pleocytosis (244 × 10^6^/L), with a predominance of mononuclear cells (98% mononuclear, 2% polymorphonuclear). Pan’s protein test was weakly positive. Immunoglobulin levels in the CSF were elevated: IgG 91.8 mg/L, IgA 14.3 mg/L, IgM 6.1 mg/L, with a total protein of 781.0 mg/L. To rule out infectious encephalitis, comprehensive multiplex pathogen testing was performed, covering bacteria, fungi, special pathogens, parasites, DNA viruses, and RNA viruses, all of which returned negative. CBA were performed on CSF and serum to detect antibodies associated with autoimmune encephalitis, including NMDAR, MOG, mGluR5, LGI1, GABAB, CASPR2, AMPA1, AMPA2, IgLON5, DPPX, GlyR1, D2R/DRD2, GAD65, mGluR1, Neurexin-3α, KLHL11, GABAA, AQP4, GFAP, and ganglionic AChR. Results showed serum positivity for NMDAR (1:30), MOG (1:100), and mGluR5 (1:10). CSF was positive for NMDAR (1:100), MOG (1:100), and mGluR5 (1:10) (**Figure 2**). These findings collectively argued against an infectious etiology and supported an immune-mediated process.

### Systemic evaluation

for occult malignancy and other organ involvement — including electrocardiogram, echocardiography, chest CT, abdominal ultrasound (liver, gallbladder, kidneys), and cervical lymph node ultrasound — was unremarkable. Notably, unlike the first reported cases of anti-mGluR5 encephalitis, this patient had no evidence of Hodgkin lymphoma. Total NK cell count was 28.78%.

### Therapeutic intervention

The patient received an aggressive multimodal immunotherapy regimen. Intravenous methylprednisolone sodium succinate was administered in a stepwise tapering fashion (500 mg/day for 3 days, followed by 250 mg/day for 3 days, then 120 mg/day for 3 days). Concurrently, intravenous immunoglobulin was given at 400 mg/kg/day for 5 consecutive days, supplemented by six sessions of plasma exchange (40 mL/kg per session). To maintain immunosuppression and reduce the risk of relapse, rituximab was administered at a dose of 100 mg once weekly for four consecutive weeks (cumulative dose: 400 mg). The patient responded favorably to the combined therapy, demonstrating significant and progressive improvement in cognitive function, psychiatric symptoms, and level of consciousness. He was discharged on oral prednisone acetate at 30 mg/day, which was to be tapered by 2.5 mg every 3 weeks.

### Follow-up and outcomes

Over a 4-month follow-up period, the patient achieved full cognitive recovery with no clinical relapses. Repeat MMSE score returned to a perfect 30/30 (**Table 1**), compared with the impaired baseline performance. Follow-up EEG normalized, and repeat brain MRI remained unremarkable. Importantly, repeat antibody testing demonstrated a meaningful serological response to treatment: serum anti-mGluR5 antibodies had turned negative, while both anti-NMDAR and anti-MOG antibody titers had declined from 1:100 to 1:10, paralleling the clinical improvement. Total NK cell count recovered to 32.83%. The patient’s clinical timeline, laboratory findings, diagnostic workup, and therapeutic interventions are summarized in **Supplementary Table S2**.

**Table 1:** Comparison of clinical and functional scores over time.

Admission (Nov 20, 2025)	Discharge (Jan 15, 2026)	4-month (May2, 2026)	Significance	
MMSE	13/30	26/30	30/30	Severe to no cognitive impairment
CASE Score*	12	2	0	Resolution of AE-specific symptoms
mRS Score	4	1	0	Return to pre-morbid disability level
Psychiatric Symptoms	Severe (Irritability, Apathy)	Resolved	Resolved	Complete psychiatric remission
Academic/Work Status	Interrupted	Preparing to return	Full-time University	Full functional reintegration

*CASE Score (Clinical Assessment Scale for Autoimmune Encephalitis) includes assessment of memory, psychiatric symptoms, language, seizures, etc.

## Discussion

Neuroimaging revealed bilateral thalamic and right hippocampal FLAIR hyperintensities, which are consistent with limbic and diencephalic involvement in NMDAR encephalitis, though less commonly reported than cortical or medial temporal abnormalities. We describe here an unusual immunological collision: the simultaneous detection of anti-NMDAR, anti-MOG, and anti-mGluR5 antibodies in a young man whose illness unfolded as a relentless erosion of memory and personality. While the pairing of NMDAR and MOG antibodies—now formally recognized as MNOS—has become a familiar entity in neuroimmunology (2, 7), the addition of mGluR5 antibodies into this mix remains virtually unprecedented, with only a single prior case documented in the literature (6). What makes our patient particularly instructive is not merely the rarity of his antibody constellation, but the clinical narrative it produced: a progressive cognitive collapse unaccompanied by seizures, and imaging findings limited to subtle hyperintensities in the bilateral thalamus and right hippocampus. This phenotype challenges our existing mental models of multi-antibody encephalitis and argues for a more nuanced understanding of how these immune attacks manifest (25).

### Navigating the diagnostic maze

The patient’s initial presentation—fever, headache, and rapidly deteriorating cognition—forced us into a diagnostic triage that felt, at times, like peeling back layers of an onion. Our first instinct, given the fever and striking CSF pleocytosis (244 cells/μL), was to treat this as infectious meningoencephalitis. We initiated empirical ceftazidime while awaiting pathogen results. Yet the patient’s clinical trajectory remained unchanged, and comprehensive multiplex PCR testing for neurotropic viruses (HSV, VZV, enteroviruses) came back negative (26). The infectious hypothesis, despite its initial appeal, simply did not hold water.

We then pivoted to consider toxic or metabolic derangements, but routine biochemical panels—renal, hepatic, thyroid—were unremarkable. The prominent behavioral changes and personality shifts might have suggested a primary psychiatric disorder, perhaps an acute psychotic break. However, the constellation of fever, an inverted CD4/CD8 ratio, elevated CSF protein, and abnormal EEG and MRI findings pointed unambiguously toward an organic, inflammatory process rather than a functional psychiatric illness (10, 25).

The prodromal infection followed by encephalopathy briefly raised the specter of acute disseminated encephalomyelitis (ADEM). Yet ADEM typically announces itself with florid, poorly demarcated demyelinating lesions scattered across the deep white matter, basal ganglia, and spinal cord. Our patient’s MRI, by contrast, showed only subtle thalamic and hippocampal hyperintensities—a pattern too focal and too selective to fit the ADEM mold (2).

The plot thickened when mGluR5 antibodies surfaced. This discovery immediately triggered concern for Ophelia syndrome, a paraneoplastic encephalitis historically linked to Hodgkin lymphoma (5). We launched an exhaustive oncological workup: chest and abdominal CT, tumor markers, cervical and axillary lymph node ultrasound. All came back clean, effectively ruling out occult malignancy.

Finally, we turned our attention to parsing the specific autoimmune encephalitis subtypes. The patient’s psychiatric and cognitive symptoms bore a strong resemblance to classic anti-NMDAR encephalitis (1, 10). Meanwhile, the presence of MOG antibodies raised the possibility of MOGAD-associated cortical encephalitis, though this entity typically features prominent seizures and cortical swelling—neither of which our patient exhibited (2). The diagnostic puzzle ultimately clicked into place when cell-based assays (CBA) confirmed triple positivity in both serum and CSF. This was not a case of mistaken identity or overlapping mimics; it was a genuine multi-antibody overlap syndrome, with MNOS serving as the foundation and mGluR5 antibodies adding an unexpected layer of complexity (6, 7).

Importantly, while the initial routine radiological report came back negative, a subsequent expert neuro-radiological review of the FLAIR sequences uncovered the subtle but telling hyperintensities in the bilateral thalamus and right hippocampus (**Figure 1**) (11). This underscores a critical lesson: in cases where clinical suspicion remains high despite unremarkable initial imaging, a second expert review can be diagnostically transformative (27). These findings, when combined with the marked CSF pleocytosis and the detection of multiple autoantibodies, left no doubt that we were dealing with an immune-mediated process.

### Decoding the antibody trio: more than the sum of its parts

To appreciate why this patient’s clinical picture diverged so sharply from typical NMDAR or MOG encephalitis, we need to consider the mechanistic interplay among the three antibodies. NMDAR antibodies are known to crosslink and internalize receptors, reducing their density on postsynaptic membranes and disrupting the delicate balance between excitation and inhibition (10, 28). This typically manifests as psychiatric symptoms, memory deficits, and seizures. Our patient certainly had the psychiatric and memory components, but the expected seizures never materialized. Electroencephalography showed only diffuse slow waves, without epileptiform discharges.

Why the absence of seizures? One possibility lies in the antibody titer distribution. Previous studies have suggested that patients with predominantly psychiatric symptoms may harbor higher CSF antibody levels than those who present with seizures or isolated memory deficits (12). In our case, the anti-NMDAR titer disparity was striking: CSF titer (1:100) far exceeded serum titer (1:30). This gradient provides definitive evidence of intrathecal synthesis (ITS), confirming that the autoimmune response was being actively generated within the CNS rather than passively leaking in from the periphery (9, 12). To enhance confidence in the serological findings—particularly given the low titers of mGluR5 (1:10) and MOG (1:100)—we employed a standardized fixed cell-based assay (CBA) and confirmed the results through repeat sampling (20, 29).

But the NMDAR antibodies were not acting alone. mGluR5 receptors are densely concentrated in the postsynaptic terminals of the hippocampus and amygdala, where they play a pivotal role in long-term potentiation (LTP), learning, memory, and behavioral regulation (13, 21). We propose that the coexistence of anti-NMDAR and anti-mGluR5 antibodies created a synergistic disruption of hippocampal synaptic plasticity—a kind of “double hit” to the memory circuitry. This synergy likely explains why our patient’s cognitive impairment was so profound, characterized by severe anterograde amnesia (an inability to form new memories) and partial retrograde amnesia, despite the relatively focal MRI lesions (11, 22).

Adding another layer of complexity, mGluR5 has been shown to regulate the differentiation of oligodendrocyte precursor cells into mature oligodendrocytes (19). Meanwhile, NMDAR and MOG antigens are co-expressed on oligodendrocyte processes (3, 8). When the immune system targets oligodendrocytes—as it does in MNOS—it may simultaneously expose both NMDAR and MOG antigens, triggering a dual autoimmune response (30). The presence of mGluR5 antibodies in this context is unlikely to be coincidental. Rather, it may reflect an amplified immune cascade triggered by the initial demyelinating insult, where myelin damage, synaptic dysfunction, and secondary immune activation feed into one another in a vicious cycle (7, 30).

### Phenotypic divergence: when antibody profiles don’t predict clinical outcomes

The only previously reported case of triple-antibody positivity (Fu et al., 2022) presented with fever, headache, and language impairment, followed by psychomotor agitation and seizures. Brain MRI showed diffuse cortical edema (6). Our patient’s trajectory was markedly different (**Table 2**). Cognitive impairment was the dominant feature, seizures were conspicuously absent, and MRI revealed only focal hyperintensities in the bilateral thalamus and right hippocampus. Moreover, our patient achieved complete clinical remission after intensive immunotherapy (methylprednisolone, intravenous immunoglobulin, rituximab, and plasma exchange), whereas the prior case had a more protracted course (6).

**Table 2:** Clinical characteristics, tumor status, treatment, and prognosis of 2 patients with MOG, NMDAR, and mGluR5 antibody-related encephalitis.

Characteristic	Rong et al. (2022)	Present case
Antibodies	NMDAR + MOG + mGluR5	NMDAR + MOG + mGluR5
Age/Sex	38/M	20/M
Clinical symptom	Fever, headache, seizures (status epilepticus), aphasia	Fever, headache, memory impairment (No seizures)
EEG	Left frontotemporal sharp/slow waves	Diffuse slow waves (4-7Hz θ), No epileptiform discharges
Brain MRI	Left hemisphere cortical edema (FLAIR/DWI hyperintensity)	Right temporal lobe and bilateral thalamus(FLAIR hyperintensity)
Tumor	None detected	None detected
Immunotherapy	IVMP + IVIG → Oral prednisone +MMF	IVMP + IVIG + Rituximab + Plasma exchange (Quadruple therapy)
Prognosis	Follow-up fou 6 months, Improved	Follow-up fou 3 months, Complete recovery
Antibodies (baseline)	NMDAR antibody CSF: 1:10, MOG antibody CSF: 1:10 S: 1:32, mGluR5 antibody CSF: 1:10 S: 1:10	NMDAR antibody CSF: 1:100 S: 1:30, MOG antibody CSF: 1:100 S: 1:100, mGluR5 antibody CSF: 1:10 S: 1:10
Antibodies (follow-up)	MOG antibody S: 1:10, other antibodies negative at 6 months	mGluR5: negative; MOG: 1:10; NMDAR: 1:10 at 3 months

M, male; IgG, Immunoglobulin G; MRI, Magnetic Resonance Imaging; T2/FLAIR, fluid attenuated inversion recovery; DWI, diffusion- weighted imaging; EEG, electroencephalogram; S, Serum; CSF, Cerebrospinal Fluid; NMDAR, N-methyl-D-aspartate receptor; MOG, myelin oligodendrocyte glycoprotein; mGluR5, metabotropic glutamate receptor 5; IVMP, Intravenous Methylprednisolone; IVIg, intravenous immunoglobulin; MMF, Mycophenolate Mofetil.

This divergence is instructive. It suggests that even when patients share a similar antibody profile, their clinical phenotypes can vary dramatically. Several factors likely contribute to this variability: antibody titers, antibody subtypes (IgG1 vs. IgG4), the topographic distribution of target antigens, the host’s underlying immune background, and the degree of blood-brain barrier disruption (9, 10, 22, 28). In our patient, the psychiatric symptoms were dominated by emotional blunting (apathy) and social withdrawal, rather than the overt hallucinations or catatonia often seen in pure anti-NMDAR encephalitis (1, 11). Although the MMSE is less sensitive for a 20-year-old student, the baseline score of 13/30 reflected profound deficits in delayed recall and executive function—a neuropsychological profile more consistent with limbic-diencephalic dysfunction than with generalized cortical hyperexcitability (18, 23).

### Immune remodeling: a nonlinear journey toward tolerance

One of the most illuminating aspects of this case was the dynamic immunological monitoring we conducted throughout the patient’s treatment and recovery. In the acute phase, the immune landscape was chaotic: the CD4/CD8 ratio was inverted, double-negative T (DNT) cells were markedly expanded, and natural killer (NK) cells were elevated. These findings pointed to robust activation of both adaptive and innate immunity—a kind of immunological “storm” raging within the CNS (10, 11, 31).

Following intensive immunotherapy, we observed a striking shift. DNT cells, which had been markedly elevated, plummeted in parallel with the patient’s clinical improvement. This temporal correlation suggests that DNT cells may serve as a real-time biomarker of disease activity—a cellular “thermometer” for neuroinflammation (31). During long-term follow-up, B cells remained depleted under rituximab treatment, while regulatory T (Treg) cells gradually increased and NK cells were reactivated. This pattern suggests a process of immune tolerance reconstruction, where the immune system transitions from a state of hyperactivation to one of regulated quiescence (24, 32).

The patient was treated with a low-dose rituximab (RTX) regimen (100 mg/week for 4 weeks, total 400 mg). While this dose is lower than oncology-based protocols, it was chosen based on evidence in Asian populations demonstrating its efficacy in achieving complete B-cell depletion while minimizing infection risks (33, 34). In our case, this regimen successfully led to profound peripheral CD19+ B-cell depletion (<10 cells/μL). Yet despite this near-complete B-cell ablation, low-titer NMDAR and MOG antibodies persisted at follow-up. This persistence likely reflects the survival of long-lived, CD20-negative plasma cells entrenched in the CNS or bone marrow—cells that are intrinsically resistant to RTX (10, 35).

### The “clinical threshold hypothesis”: when seropositivity doesn’t mean pathogenicity

Perhaps the most intriguing observation from this case was the “clinical-immunological dissociation” we observed at the 4-month follow-up. The patient achieved complete neurological recovery, and mGluR5 antibodies became undetectable. Yet NMDAR and MOG antibodies remained positive, albeit at reduced titers (66.7% and 90% decreases from baseline, respectively). This dissociation challenges the conventional wisdom that clinical remission requires complete seronegativity (12, 36).

We tentatively propose a “clinical threshold hypothesis” to explain this phenomenon. In autoimmune encephalitis with multiple coexisting antibodies, persistent seropositivity does not necessarily indicate ongoing pathogenicity. Rather, clinical recovery may occur once antibody titers fall below an individual-specific pathogenic threshold—a tipping point below which the antibodies’ disruptive effects on neural network function are offset by compensatory mechanisms. Think of it as a dimmer switch rather than an on-off toggle: as antibody levels decline, their impact on synaptic function gradually fades, even if they never fully disappear (11, 12, 36).

This framework has practical implications. It suggests that treatment decisions—particularly around maintenance therapy or drug tapering—should not rely solely on achieving complete antibody negativity. Instead, clinicians should integrate clinical presentation, dynamic immunological changes (such as Treg recovery and normalization of the CD4/CD8 ratio), and the patient’s individual relapse risk profile (10, 24, 32). However, we emphasize that this is a hypothesis-generating observation from a single case, and further validation in larger cohorts is needed.

### Contextualizing our case within the MNOS literature

MNOS is the most commonly reported form of neural autoantibody overlap syndrome (7, 14). A systematic review by Saeed et al. included 256 patients with anti-NMDAR encephalitis and demyelinating syndromes, with MOGAD accounting for 94.5% (approximately 242 cases) (16). A retrospective study by Ma et al. analyzed 23 adult patients with MNOS and found a strong male predominance (86.96%), a mean age at onset of 36.17 years, and a clinical profile dominated by sleep disturbance (69.57%), psychiatric and cognitive symptoms (65.22%), impaired consciousness (60.87%), and seizures (56.52%) (17). Our patient—a young male with prominent psychiatric disturbance and profound cognitive decline—fits this demographic and clinical pattern.

Importantly, Ma et al. reported a relapse rate of approximately 26.09%, suggesting that persistent MOG antibodies may increase the risk of long-term recurrence (17, 24). This underscores the need for vigilant follow-up in our patient, with close monitoring for both demyelinating events and recurrent encephalitic symptoms. Given that MOG antibodies remained positive (1:10) at the 4-month follow-up, the patient remains at risk for clinical relapse (15). Continued long-term surveillance is mandatory to determine whether the reconstruction of immune tolerance—indicated by the recovery of Treg cells and normalization of the CD4/CD8 ratio—is durable enough to prevent future episodes (24, 32).

### Limitations and the path forward

Several caveats temper our interpretation of this case. First, the low titer of anti-mGluR5 antibodies (1:10) and the absence of functional validation studies (e.g., live neuron-based assays, antibody transfer experiments) limit our ability to definitively establish pathogenicity (22, 29). The possibility of false positivity or clinically insignificant bystander antibodies cannot be entirely excluded. Second, we did not perform antibody subtyping (IgG1 vs. IgG4), which might have provided deeper insights into pathogenic mechanisms (22). Third, the absence of tissue-based assays (e.g., rat brain immunohistochemistry) as confirmatory testing represents a technical limitation (29). Fourth, we cannot rule out that the rapid seroreversion of mGluR5 antibodies was coincidental rather than causally related to treatment. Finally, the relatively short 4-month follow-up is insufficient to declare a definitive cure, especially given the notorious relapsing nature of MOG-associated diseases (15, 17, 24).

Despite these limitations, the comprehensive clinical, immunological, and longitudinal data we present offer valuable insights into this rare overlap syndrome. This case not only expands the clinical spectrum of MNOS but also provides a window into the dynamic, nonlinear process of immune reconstitution. It suggests that healing from these devastating attacks is not a simple matter of antibody clearance, but rather a complex rebalancing of immune networks—a process where clinical functionality can vastly outpace complete seronegativity. As we navigate the complexities of long-term care in such patients, prioritizing the clinical trajectory alongside cellular immune reconstitution appears to be a far more rational approach than chasing an elusive seronegative state, provided that rigorous vigilance for relapses and occult malignancies is maintained.

## Conclusions

We reported a rare case of an overlapping syndrome with the coexistence of MOG-IgG, NMDAR-IgG and mGluR5-IgG, which widens the understanding of this disease.The case featured marked DNT cell expansion and persistent plasma cell-derived autoantibody production, along with a cognitive-dominant phenotype without seizures. These findings broaden the clinical spectrum and highlight the importance of individualized immunotherapy and immune monitoring.

## Data Availability

The raw data supporting the conclusions of this article will be made available by the authors, without undue reservation.
