# Supplementary material for: Triple antibody-associated autoimmune encephalitis overlap: a case report of co-existing MOG-IgG, anti-NMDAR, and anti-mGluR5 positivity
Source: Front Immunol. 2026 May 22;17:1848797. doi: 10.3389/fimmu.2026.1848797 (PMC13236903; doi:10.3389/fimmu.2026.1848797)
Supplement: Supplementary file 1 [file Image1.pdf]

Supplementary TABLE S1. Longitudinal profile of key immunological parameters correlated with clinical course and treatment phases.

**Cell Subset**	**Marker**	**Reference range (%)**	**Day 35**	**Day 66**	**Day 109**
CD4/CD8	CD3+CD4+ / CD3+CD8+	1.0–4.9	0.60↓	1.04	0.67↓
Total B (%)	CD3+	53.7–82.8	—	0.01↓	0.00↓
DNT(%)	CD3+CD4−CD8−	1-5	31.10	6.62	19.62
Treg cell(%)	CD3+CD4+CD25highCD127low	5.1–12.7	6.70	8.08	9.68
NK Cells(%)	CD3− CD16+CD56+	3.3–32.9	28.78	5.07	32.83
CD4+ T helper(%)	CD3+CD4+	46.2–78.0	25.72↓	47.48	32.21↓

Notes:

Data are presented as percentages of positive cells within the indicated lymphocyte gate. Arrows (↓) indicate values below the lower limit of the reference range. A dash (—) indicates not tested or insufficient sample.

Abbreviations: DNT, double-negative T cells (CD3+CD4−CD8−); Treg, regulatory T cells (CD3+CD4+CD25highCD127low); NK, natural killer cells.

Reference range: Based on age-matched healthy controls from the institutional laboratory.

Supplementary TABLE S2. Longitudinal clinical, diagnostic, therapeutic, and immunological timeline of the case.

Date (2025-2026)	Day	Category	Event / Investigation / Treatment	Result / Clinical Reasoning
Nov 7, 2025	0	Onset	clinical signs	Fever,headache
Nov 20, 2025	13	Admission	Clinical Presentation	MMSE 13/30; WBC 18.27×10^9/L (Ref: 4.0-10.0)
Nov 21, 2025	14	Imaging	Brain MRI	Bilateral thalami FLAIR hyperintensity
Nov 21, 2025	14	Neurophys.	EEG	Diffuse theta slowing (4–7 Hz); no epileptiform discharge
Nov 22, 2025	15	CSF	Lumbar Puncture	Pressure: 180 mmH_2O(80-180);WBC: 244 cells/μL(0-5);Protein: 781 mg/L(150-450)
Nov 25, 2025	18	Diagnosis	Antibody CBA (1st)	NMDAR (CSF 1:100), MOG (1:100), mGluR5 (1:10)
Nov 26 - Dec 6	19	Tx: Mode 1	IV Methylprednisolone	Pulse therapy: 500mg(3d) -250mg(3d) -120mg(3d)
Nov 27, 2025	20	Diagnosis	Antibody CBA (2nd)	Confirmed mGluR5 1:10 (Verification of low-titer)
Nov 26 - Dec 2	25	Tx: Mode 2	IVIG	400 mg/kg/day for 5 days
Dec 2,2025	32	Tx: Mode 4	Rituximab	100mg weekly
Dec 12, 2025	35	Monitoring	B-cell Monitoring	Memory B cells (CD19+CD27+)0.04%↓

Date (2025-2026)	Day	Category	Event / Investigation / Treatment	Result / Clinical Reasoning
Nov 7, 2025	0	Onset	clinical signs	Fever,headache
				(0.6–6.3%)
Dec 19 - Dec 30	42-53	Tx: Mode 3	Plasma Exchange (PLEX)	6 sessions (q2d); Started 20d after IVIG to avoid IVIG washout
Jan 12, 2026	66	Monitoring	B-cell Monitoring	Memory B cells (CD19+CD27+)0.01%↓ (0.6–6.3%)<br>Total B cells 0.01↓(3.8–21.5)
Jan 13, 2026	67	Imaging	Brain MRI	Bilateral thalamic & right hippocampal FLAIR hyperintensity<br>bilateral thalami FLAIR
Feb 24,2026	109	Monitoring	B-cell Monitoring	Memory B cells (CD19+CD27+)0.00%↓ (0.6–6.3%)<br>Total B cells 0.00↓ (3.8–21.5)
Feb 25,2026	110	Imaging	Brain MRI	Bilateral thalamic & right hippocampal FLAIR decreased signal intensities
Mar 20, 2026	134	Follow-up	Serology & Imaging	MMSE 30/30; mGluR5 negative; MRI lesions resolved

This table aligns clinical course , therapeutic interventions, and immune marker dynamics along a unified time axis (Day 0–134). Key time points (Day 35, 66, 109) correspond to those in Supplementary Table S1.

Abbreviations: CBA, cell-based assay; CSF, cerebrospinal fluid; EEG, electroencephalography; FLAIR, fluid-attenuated inversion recovery; IVIG, intravenous immunoglobulin; MMSE, Mini-Mental State Examination; MOG, myelin oligodendrocyte glycoprotein; mGluR5, metabotropic glutamate receptor 5; MRI, magnetic

resonance imaging; NMDAR, N-methyl-D-aspartate receptor; PLEX, plasma exchange; Tx, treatment; WBC, white blood cell. ↓ indicates below reference range. Reference ranges are shown in parentheses. Treatment modes: 1 = IV methylprednisolone; 2 = IVIG; 3 = plasma exchange; 4 = rituximab.
